# Effect of levetiracetam on ocular perfusion measure with optical coherence tomography angiography

**DOI:** 10.5935/0004-2749.2022-0269

**Published:** 2023-09-27

**Authors:** Ahmet Kaderli, Hülya Kayılıoğlu, Sema Tamer Kaderli, Aylin Karalezli

**Affiliations:** 1 Ophthalmology Department, Mugla Sitki Kocman University Research and Training Hospital, Muğla, Turkey; 2 Pediatric Neurology Department, Mugla Sitki Kocman University Research and Training Hospital, Muğla, Turkey

**Keywords:** Intraocular pressure, Levetiracetam, Tomography, optical coherence, Fluorescein angiography, Epilepsy, Child, Pressão intraocular, Levetiracetam, Tomografia de coerência óptica, Angiofluoresceinografia, Epilepsia, Criança

## Abstract

**Purpose:**

To evaluate using optical coherence tomography angiography the macular and optic nerve head blood flow in pediatric patients with epilepsy treated with levetiracetam for at least 12 months.

**Methods:**

This study included 33 pediatric patients with epilepsy and 30 sex- and age-matched healthy volunteer children were included in the study. Optical coherence tomography angiography was used to evaluate the optic nerve head and macular perfusion changes. The mean ocular perfusion pressures were also calculated. Patients who were using multiple antiepileptic drugs or had a prior history of using different drugs were excluded.

**Results:**

The choriocapillaris flow area was significantly lower in the Study Group than in the Control Group (p=0.006). However, the foveal avascular zone and vessel densities of the macula in the superficial capillary plexus, deep capillary plexus, and optic nerve head of the study group were not significantly different from those of the control group (p>0.05). Moreover, no significant difference in means of mean ocular perfusion pressure was found between the two groups (p=0.211). No obvious correlation was found between treatment duration and optical coherence tomography angiography parameters or mean ocular perfusion pressure.

**Conclusion:**

Choroidal perfusion was reduced in children taking levetiracetam compared with that in the control group, whereas retinal perfusion was not affected in this optical coherence tomography angiography study.

## Abstract (pt)

**Objetivo:**

Avaliar através de angiotomografia de coerência óptica o fluxo sanguíneo macular e da cabeça do nervo óptico em pacientes pediátricos com epilepsia tratados com levetiracetam por pelo menos 12 meses.

**Método:**

Trinta e três pacientes pediátricos com epilepsia e 30 crianças voluntárias saudáveis pareadas por sexo e idade foram incluídos no estudo. A angiotomografia de coerência óptica foi utilizada para avaliar as alterações da perfusão da cabeça do nervo óptico e da macular. As médias das pressões de perfusão ocular também foram calculadas. Pacientes em uso de múltiplas drogas antiepilépticas ou com história prévia de uso de diferentes drogas foram excluídos do estudo.

**Resultado:**

A área do fluxo coriocapilar foi significativamente menor no Grupo Estudo do que no Grupo Controle (p=0,006). Entretanto, a zona avascular foveal e as densidades vasculares no plexo capilar superficial e profundo da região macular e na cabeça do nervo óptico não foram significativamente diferentes daquelas de olhos saudáveis (p>0,05). Também não houve diferença significativa entre os dois grupos em relação às médias da pressão de perfusão ocular (p=0,211). Nenhuma correlação aparente foi encontrada entre a duração do tratamento e os parâmetros da angiotomografia de coerência óptica ou a média da pressão de perfusão ocular.

**Conclusão:**

Em crianças usando levetiracetam, a perfusão coroidal mostrou-se reduzida em comparação ao grupo controle, enquanto a perfusão retiniana não foi afetada neste estudo com angiotomografia de coerência óptica.

## INTRODUCTION

Epilepsy is a common neurological disorders in the pediatric population and an important reason for disability and mortality^(1)^. Up to 1% of children are suffering from epilepsy^(2)^. Epilepsy is described as recurrent seizures induced by abnormal neural activity in the brain^(3)^. Many antiepileptic drugs with varying effect mechanisms can be used in the treatment^(4)^.

In the last few decades, new drugs such as levetiracetam, felbamate, gabapentin, lamotrigine, pregabalin, topiramate, and vigabatrin are used in the treatment of epilepsy. Ocular side effects of these antiepileptic drugs include blurred vision, visual field defects, color disturbances, diplopia, nystagmus, retinopathy, and maculopathy^(5,6,7,8)^. A study suggested that vigabatrin, by blocking gamma-aminobutyric acid (GABA) transaminase, decreases cerebral and ocular blood flow by increasing GABA levels, and this decrease may affect the vitality and functioning of the neuroretina, resulting in decreased retinal sensitivity and visual performance^(9)^.

Levetiracetam is a broad-spectrum antiepileptic drug that has been approved in children aged >1 month ^(10)^. It can be used as adjunctive therapy for focal and generalized epilepsy in children^(11)^. Its mechanism of action is the modulation of synaptic neurotransmitter release by binding to the synaptic vesicle protein SV2A in the brain. Levetiracetam influences the possible mechanism associated with the blockage of zinc and beta-carbolines by preventing chloride entrance in GABA and glycine receptors^(12,13)^. As it is a relatively new drug, studies on its ocular side effects are limited, and its effects on retinal anatomy and physiology are unknown.

Optical coherence tomography angiography (OCTA) is a novel noninvasive, repeatable, and high-resolution imaging technique that visualizes the retina and choroidal microvascular structures in the macula, optic nerve head (ONH), and peripapillary areas^(14)^.

We hypothesized that levetiracetam might have potential ocular side effects, as levetiracetam indirectly facilitates the inhibitory effects of GABA at synapses, similar to vigabatrin and other antiepileptics^(15)^. In this study, we aimed to evaluate the macular and ONH blood flow via OCTA in pediatric patients with epilepsy who have been on levetiracetam treatment for at least 12 months.

## METHODS

The study observational cross-sectional study was approved by the Mugla University School of Medicine Ethical Committee (No. 13/11, 11.11.2020). All patients met the eligibility criteria, and written informed consent from the patients and their parents was obtained.

### Patients and subjects

This study included 33 pediatric patients with epilepsy who were followed up in the pediatric neurology department and on levetiracetam monotherapy for at least 12 months at a dosage of 15-25 mg/kg/day (group 1, Study Group). Moreover, 30 sex- and age-matched healthy volunteer children who were followed up in the ophthalmology department were also enrolled (group 2, Control Group).

Electroencephalography outcomes were classified as focal, generalized, or normal in the Study Group. All participants underwent comprehensive ophthalmological examinations including the best-corrected visual acuity, intraocular pressure measurement, biomicroscopic slit-lamp examination, and OCTA. The exclusion criteria were as follows: patients using multiple antiepileptic drugs or prior history of different drug use, uncooperative children because of mental retardation or young age, participants (Study or Control Group) with cycloplegic refractive error over three diopters spherical equivalent (to reduce the effects of refractive changes on the results), patients with retinal vascular diseases, any kind of nystagmus and amblyopia, a history of previous ocular surgery, history of glaucoma or non-glaucomatous optic neuropathies, any media opacity limiting acceptable image quality, and image quality under 8/10 because of fixation inabilities.

## OCTA

**Figure 1:**
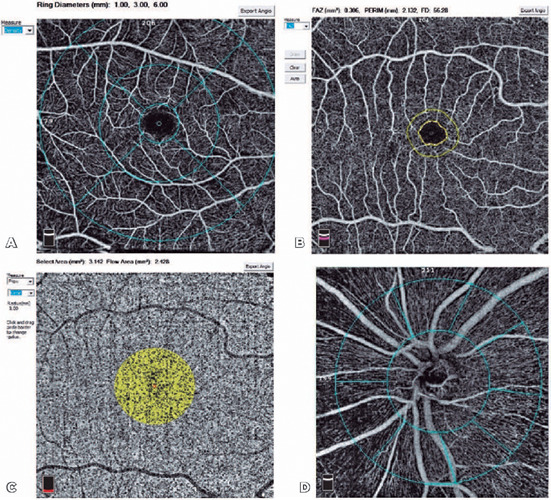
(A) Angiomacula (fovea, parafovea, and perifovea) in 6 × 6 mm scan size. (B) Foveal avascular zone. (C) Choroidal capillary plexus fow area in 1-mm radius circle. (D) Vessel densities for the optic disc at a 4.5 × 4.5-mm scan size.

**Figure 2:**
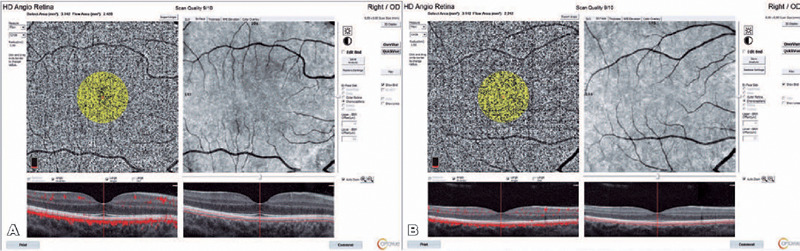
Choroidal capillary plexus fow area (A) in a Study Group participant and (B) a Control Group participant.

All images were captured with the AngioVue Imaging System (RTVue XR Avanti; Optovue, Inc., Fremont, CA, USA) by the same qualified observer. The AngioVue Imaging System is a spectral-domain OCT device that supports concurrent three-dimensional anatomical imaging of the retina and produces en face projections of the blood flow within a split-spectrum amplitude-decorrelation angiography algorithm. The AngioAnalytics software (Optovue, Inc.) provides the area of the foveal avascular zone (FAZ) and capillary vessel density (VD) from the selected areas of the retina. Quantitative analysis of 6 × 6 mm OCT angiograms was taken for the automated detection of flow including the FAZ (mm²), capillary VD (%), and choriocapillaris flow area (mm²) analysis. The device automatically embedded three fovea-centered concentric circles on the macula by a density estimation device in both superficial capillary plexus (SCP) and deep capillary plexus (DCP) (Figure 1A). The VD was automatically calculated by the OCTA scanner. The SCP was located between 3 µm below the inner limiting membrane and 15 µm below the inner plexiform layer, and the DCP extended from 15 µm to 70 µm below the inner plexiform layer. The foveal zone VD was determined by the area of the inner circle with a diameter of 1 mm. The area of the middle circle with a diameter of 3 mm was determined as the parafoveal zone VD, and the area of the outer circle with a diameter of 6 mm was defined as the perifoveal zone VD. The flow area of the choriodal capillary plexus (CCP), which was centered on the FAZ, was collected at 1-mm radius areas and calculated by the number of pixels over the threshold from the en face OCTA (Figure 1B, 1C and Figure 2). The peripapillary flow was validated by the total and peripapillary flow, and the inside-disc VD was measured using a 4.5 × 4.5 mm scan that was centered on the ONH. The device automatically hangs a 2-mm diameter circle centered on the optic disc and determines the peripapillary area as a 1 mm-wide ring annulus spreading from the optic disc 2-mm circle. The peripapillary vessels were analyzed in the radial peripapillary capillary section that prolongs from the inner limiting membrane to the retinal nerve fiber layer. The peripapillary VD was expressed as the percentage area filled by microvasculature in the peripapillary area. VDs for the whole 4.5 × 4.5 mm scan region (whole image), optic disc area (inside disc), and entire peripapillary area were determined by applying an automated software algorithm. Peripapillary VD, which was represented as the whole width of the perfused vasculature per unit distance in the field of analysis, was automatically included in the mean outcome (Figure 1D). OCTA images as Q8 or higher quality were accepted.

The mean ocular perfusion pressure (MOPP) was calculated following the equation described by Van Keer et al.^(16)^.

$$MOPP:\frac{2}{3}\times \text{mean arterial pressure}-\text{intraocular pressure}$$

The MOPP and OCTA parameters were compared between pediatric patients with epilepsy under levetiracetam treatment and healthy volunteers.

### Statistical analysis

Data analyses were performed using IBM SPSS Statistics version 23.0 (Armonk, NY, USA). The distribution of continuous variables (normal or not normal) was determined using the Shapiro-Wilk test. The Levene test was used to evaluate the homogeneity of variances. Continuous data were described as the mean ± standard deviation (SD) for normal distributions and median (range) for skewed distributions. The variations between normally distributed continuous variables among independent groups were analyzed with Student’s t-test. The chi-squared test or Fisher’s exact test was used to analyze categorical variables and compare demographic factors between pediatric patients with epilepsy and healthy controls. Pearson’s correlation coefficient was used to demonstrate the relationship between changes in treatment duration against OCTA parameters and MOPP. P<0.05 was considered statistically significant.

## RESULTS

No significant difference in age, sex, laterality and spherical equivalent of refractive error, intraocular pressure, and mean arterial pressure was found between the two groups (p>0.05 for all). Table 1 shows the demographic and clinical characteristics of all participants. The mean treatment duration was 24.95 ± 13.52 months for the Study Group.

**Table 1:** Baseline and clinical characteristics of the study and control groups

		Study group	Control group	p-value
Age, months (mean±SD)		139.57 ± 37.75	131.60± 37.60	0.405^*^
Sex, F/M (%)		25/8 (75.7/24.3)	25/5(83.3/16.7)	0.542^**^
Laterality, R/L (%)		16/17 (50/50)	15/15(50/50)	0.904^**^
Spherical equivalent of refractive error, D		*0.83 ± 0.32	-0.15 ± 0.32	0.423^*^
IOP		12.15 ± 1.22	12.00 ± 1.70	0.685^*^
MAP		77.69 ± 7.41	80.20 ± 9.07	0.233^*^
EEG type, normal/focal/generalized (%)		1/18/4 (33.3/54.5/12.1)	-	-
Levatiracetam dosage, n (%)	15 mg/kg/day	2 (9.1)	-	-
	20 mg/kg/day	18 (81.8)	-	-
	25 mg/kg/day	2 (9.1)	-	-

D= diopters; EEG= electroencephalogram; IOP= intraocular pressure; MAP= mean arterial pressure; SD= standard deviation.

*Student’s t-test, **Fisher’s exact test.

The mean of CCP flow areas were 2.229 mm^2^ and 2.303 mm^2^ in the Study and Control groups, respectively, and the CCP flow area was significantly lower in the Study Group (p=0.006). However, the FAZ, VDs of the macula in the SCP, DCP, and ONH were not significantly different from those of the healthy eyes (Table 2). No significant difference in means of MOPP was found between the two groups (p=0.211).

**Table 2:** Comparison of OCTA parameters and MOPP between the study and control groups

		Mean ± SD		
		Study group	Control group	p-value
FAZ, mm^2^		0.315±0.09	0.323±0.10	0.754
VD in the SCP, %	Fovea	21.8±5.04	19.5±4.68	0.066
	Parafovea	54.3±3.21	54.4±3.24	0.915
	Perifovea	51.7±1.93	52.5±2.17	0.102
VD in the DCP, %	Fovea	38.3±6.44	36.1±6.57	0.187
	Parafovea	59.7±3.43	60.5±3.60	0.388
	Perifovea	58.3±3.92	59.3±3.47	0.309
VD in the ONH, %	Peripapillary	50.1±2.62	51.0±3.74	0.320
	Total disc	49.1±1.80	50.1±2.36	0.058
	Inside disc	52.8±3.95	54.2±4.87	0.320
CCP flow area, mm^2^		2.229±0.115	2.303±0.084	**0.006**
MOPP		39.7	41.4	0.211

CCP= choriocapillary plexus; DCP= deep capillary plexus; MOPP= mean ocular perfusion pressure; SCP= superficial capillary plexus; SD= standard deviation; VD= vessel density. The bold value is statistically significant (p<0.05).

The relationship between the duration of levatirecetam treatment and microvascular parameters detected by OCTA was evaluated, and no significant correlation was found between the treatment duration and OCTA parameters or MOPP (Table 3).

**Table 3:** Correlation between treatment duration and OCTA parameters and MOPP

	Treatment duration	
	r	p-value
FAZ	0.189	0.400
Parafoveal VD in the SCP	0.105	0.643
VD in the DCP	−0.117	0.603
Peripapillary VD	0.024	0.916
Whole-disc VD	−0.077	0.735
Inside-disc VD	0.311	0.158
CCP flow area	−0.018	0.937
MOPP	0.119	0.599

CCP= choriocapillary plexus; DCP= deep capillary plexus; FAZ= foveal avascular zone; SCP= superficial capillary plexus; VD= vessel density.

## DISCUSSION

This study showed that OCTA findings are comparable in most parameters in children diagnosed with epilepsy who were using levetiracetam compared with the healthy population. This may indicate that the long-term drug use does not affect ocular perfusion. The similarity in MOPP results as in OCTA findings between the two groups may further strengthen the interpretation on this issue.

Vigabatrin is used in the treatment of infantile spasm in children, especially in patients diagnosed with tuberous sclerosis, and in adjunctive therapy in drug-resistant focal epilepsy. It is used cautiously because of its ocular side effects. Thus, ocular side effects must be monitored during drug use. Vigabatrin is contraindicated in the case of adverse effects on the visual field. Jonsson et al. evaluated the effects on long-term visual field in children who received vigabatrin and found the risk of developing ocular side effects at a rate of 31% with the use of vigabatrin in children for more than 6 months^(17)^. Biswas et al. reported retinal toxicity rate of 29% in patients who were diagnosed with epileptic spasm and were using vigabatrin ^(18)^. In the meta-analysis by Maguire et al., visual field defects developed in 34% of pediatric patients with focal epilepsy after the use of vigabatrin^(19)^.

In addition to more frequently reported side effects such as visual field loss and color vision deficit^(20)^, some antiepileptics have been shown to reduce ocular blood flow^(21)^. Hoskin et al. explained this effect through several mechanisms. The first possibility was that the effect of antiepileptics on cerebral blood flow may be mediated by increased GABA levels in the cerebrospinal fluid, which is known to lower the metabolic rate for glucose and blood flow^(22)^. Vigabatrin increases GABA levels more than other antiepileptics, and more inactivation is observed in the retina than in the brain^(23)^. Another possible explanation was pre-existing ischemia in patients with epilepsy^(21)^.

The mechanism action of levetiracetam is the modulation of synaptic neurotransmitter release by binding to the synaptic vesicle protein SV2A in the brain. Studies have shown that the drug influences the possible mechanism associated with the blockage of zinc and beta-carbolines by preventing chloride entrance in GABA and glycine receptors^(12,13)^. The drug acts by a mechanism different from vigabatrin and works independent of GABA inhibition. In this study, levetiracetam did not show any serious side effects on ocular perfusion because of the different mechanism of action.

Levetiracetam is a commonly used new-generation antiepileptic drug and has been previously studied for its ocular side effects. Retinal nerve fiber layer thickness, macular ganglion cell complex, central cornea, or foveal thickness values were not different when measured with OCT in pediatric patients with epilepsy receiving leve-tiracetam therapy compared with those in the healthy population. None of the patients had impaired color vision nor visual field defect^(24)^. Although levetiracetam appears to be safe considering its effects on the eyes, Hazirolan et al. showed that central macular and ganglion cell complex thicknesses and visual evoked potential (VEP) parameters in patients using levetiracetam may differ from those in healthy controls. They reported thinner central macular thickness in OCT, prolonged latency of N135, and decreased P100 amplitude in VEP^(25)^. When we evaluated the effect of the drug on ocular perfusion, the Study Group and the Control Group revealed comparable OCTA parameters, except for the CCP flow area, and the treatment duration did not affect the results.

The CCP flow area was smaller in the Study Group than in the Control Group. The choriocapillaris layer provides oxygen and metabolic exchange to the outer retina, including retinal pigmentation epithelium and photoreceptors^(26)^. The retinal circulation accounts for nearly 15% of the metabolic activity of the photoreceptor inner segments in a healthy person^(27)^. When the choroidal circulation is suppressed because of hypoxia, the oxygen supply from the retinal circulation to the outer layer increases^(28)^. This indicates that retinal and choroidal circulation work together to meet the metabolic needs of the photoreceptors. However, the choroidal circulation is essential to photoreceptor health. The flow areas of the CCP, which were lower in the Study Group, appeared to be the early negative effect of drug use on ocular perfusion, Contrastingly, it may have arisen because the OCTA device used in our study did not work with the swept-source mechanism. Swept-source OCTA devices have a longer wavelength and decreased sensitivity roll-off, following improved light penetration throughout the RPE and more reliable detection of signals from the more profound layers^(29,30)^.

The estimated MOPP was reported to have no association with OCTA vessel densities^(31)^. This irrelevance was explained with the autoregulation of ocular blood flow by the variation in vascular resistance^(32)^. This may indicate that ocular blood flow depends not only on ocular perfusion pressure but also on vascular resistance^(33)^. In this study, both OCTA VD parameters and MOPP values did not differ with the use of levetiracetam when compared with the healthy pediatric population.

The main study limitation is the cross-sectional design. The absence of baseline OCTA values for the Study Group, and the results were not evaluated with repeated measurements, which may contradict the relevance of the results only to the associated drug. Future randomized controlled trials will shed light on more detailed answers on the topic. Besides being a new technique and its limitations, OCTA has already known artifacts. Various image-capturing and data-analyzing techniques may present very inconsistent outcomes. Allegrini et al.^(34)^ showed that different projection maps can demonstrate small vascular structures, which cannot be seen in common projections. Therefore, a careful approach must be considered when comparing the outcomes of different studies.

To the best of our knowledge, this is the first study to report ocular perfusion in pediatric patients who were diagnosed with epilepsy and were using levetiracetam with long-term follow-up. Double-blinded randomized reports involving a comprehensive patient population are still needed to confirm that levetiracetam is safe for ocular perfusion.
